# Acute Necrotizing Encephalitis as an Early Manifestation of COVID-19

**DOI:** 10.7759/cureus.27928

**Published:** 2022-08-12

**Authors:** Sachin Gadani, Adam Cohen

**Affiliations:** 1 Neurology, Johns Hopkins University, Baltimore, USA; 2 Neurology, Johns Hopkins University Applied Physics Laboratory, North Laurel, USA

**Keywords:** therapeutic plasmapheresis, covid19, sars-cov-2, parainfectious encephalitis, acute necrotizing encephalitis

## Abstract

In addition to respiratory symptoms, SARS-CoV-2 infection has been linked to numerous neurologic sequelae including acute necrotizing encephalopathy. Here we present the case of a 33-year-old woman infected with SARS-CoV-2 who arrived at the hospital unresponsive. She was comatose with intact brainstem reflexes, and brain imaging was consistent with acute necrotizing encephalopathy affecting the bilateral thalami, medial temporal lobes, and pons. She was treated quickly with intravenous corticosteroids and plasmapheresis and regained neurologic function over weeks. Acute necrotizing encephalopathy is a rare para-infectious syndrome characterized by rapidly progressing encephalopathy, seizures, and/or coma caused by multifocal inflammatory central nervous system (CNS) lesions. The mechanism(s) underlying this condition remain unclear, though cytokine storm and disruption of the blood-brain barrier has been proposed as initiating event. This report presents a case of adult acute necrotizing encephalopathy in the early period after SARS-CoV-2 infection, adding to the literature on this rare condition and its relation to SARS-CoV-2 infection. We also report on the clinical outcome of treatment with prompt immunosuppression.

## Introduction

The global coronavirus disease 2019 (COVID-19) pandemic has led to millions of infections with SARS-CoV-2 and unprecedented morbidity and mortality. Neurologic complications of COVID-19 infection include encephalopathy, anosmia, cerebrovascular disease, acute disseminated encephalomyelitis (ADEM), and Guillain-Barré syndrome (GBS) [1], and are becoming increasingly clinically relevant given the evolving and widespread nature of the pandemic.

Acute necrotizing encephalopathy (ANE) is a para-infectious autoimmune condition that typically affects children suffering from influenza, but has also been reported in adults and related to other viral infections [2,3]. ANE occurs within days to weeks of viral symptoms and fever and is often characterized by rapid progression to coma in the absence of cerebrospinal fluid (CSF) pleocytosis. Imaging typically shows bilateral symmetric involvement of the thalamus and deep brain structures with areas of necrosis and microhemorrhage within lesions [2]. ANE is associated with high mortality, in part due to delayed diagnosis given its rarity and often delayed imaging in clinically unstable patients. Early immunosuppression with intravenous (IV) corticosteroids, IVIg, and/or plasmapheresis appears to improve outcomes, but the optimal treatment paradigm remains unclear [3,4]. ANE has been associated with SARS-CoV-2 infection in adults [5-11] and children [12,13]. Here, we present the case of a young woman with COVID-19 who developed ANE atypically early after infection and describe her initial response to treatment and further course.

## Case presentation

A 33-year-old unvaccinated woman with pre-diabetes and a history of tobacco use was brought to the hospital after being found unresponsive. Three days before arrival she developed throat pain, fever, and sinus congestion without any reported neurologic symptoms, and the day before arrival she tested positive for SARS-CoV-2. She was febrile, tachycardic, and had leukocytosis to WBC 13.5 K/mL. On exam, she had her eyes closed and was unresponsive to voice. She had preserved corneal, pupillary light, gag, and cough reflexes. There were no spontaneous movements, but there was grimace to painful stimulus in the bilateral upper extremities and withdrawal in the bilateral lower extremities. Deep tendon reflexes were intact in the arms and legs. She was intubated for airway protection. Chest imaging revealed left lower lobe pneumonia, and she was treated with remdesivir.

**Figure 1:**
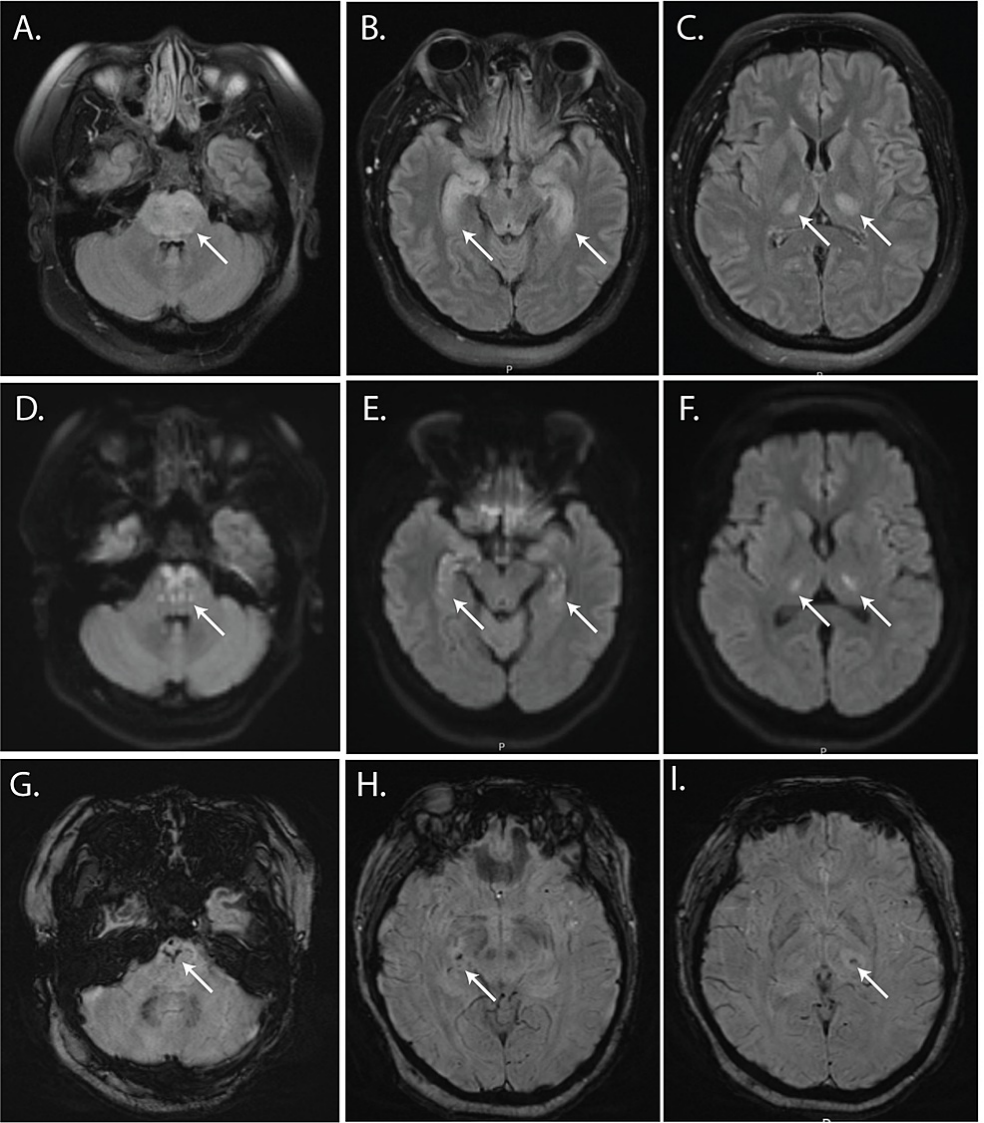
MRI scan on arrival at the hospital. Axial images from MRI obtained on admission showing T2 FLAIR (A-C), diffusion-weighted imaging (DWI) (D-F), and susceptibility weighted imaging (SWI) (G-I) sequences. T2 FLAIR hyperintense lesions contain punctate DWI signal and areas of susceptibility artifact (white arrows).

Computed tomography (CT) scan of the head demonstrated hypodensity in the brainstem and bilateral thalami without hemorrhage. Brain magnetic resonance imaging (MRI) scan showed abnormal areas of T2 hyperintensity in the pons, medial temporal lobes, and thalami (Figure 1A, 1B, 1C) with corresponding areas of diffusion restriction (Figure 1D, 1E, 1F) and susceptibility artifact (Figure 1G, 1H, 1I), consistent with ANE. Serum interleukin (IL)-6 was not elevated. Cerebrospinal fluid (CSF) opening pressure was elevated to 35cm H20. CSF analysis showed WBC 5 K/mL, RBC 0 K/mL, glucose 99 mg/dL (serum glucose 155 mg/dL), and protein 147mg/dL.

**Figure 2:**
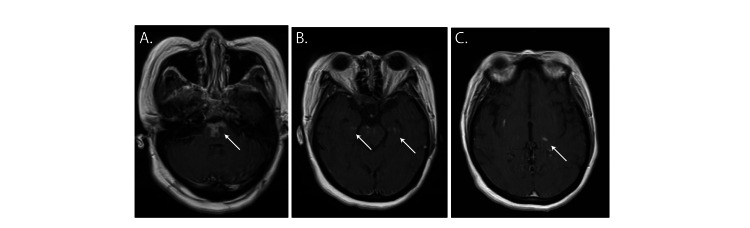
MRI scan two weeks after admission shows contrast enhancement. Axial T1 post-contrast images (A-C) show areas of contrast enhancement in the pons, mesial temporal lobes, and thalami (white arrows).

She was treated with plasmapheresis, five total treatments administered every other day, and IV methylprednisolone, 1g daily for five days, followed by a four-week oral prednisone taper. Repeat MRI two weeks after admission showed interval resolution of T2 hyperintensity in all affected brain areas and new punctate contrast enhancement in areas of prior restricted diffusion (Figure 2). She experienced gradual neurologic improvement. Three weeks after arrival, she was discharged awake, interactive, and following simple commands. She had poor attention, was unable to follow multistep commands, displayed right/left confusion, and had persistent proximal weakness in her arms and legs. 

She was discharged to inpatient rehab three weeks after admission, where she initially continued to have gradual neurologic improvement. She was persistently short of breath, and two weeks after discharge had a hypoxemic cardiac arrest and was resuscitated and readmitted to the hospital. She ultimately suffered a second cardiac arrest attributed to respiratory failure and died about two months after SARS-CoV-2 symptom onset.

## Discussion

A diverse range of neurologic complications have been reported following COVID-19, including anosmia, cerebrovascular disease, Guillain-Barré syndrome, encephalitis, and myelitis [14]. While ANE is certainly an uncommon sequela of COVID-19, the exact risk is difficult to estimate, in part because of underdiagnosis and underreporting. One recent literature review identified 23 published adult cases of COVID-19-related ANE, most of which occurred in patients without prior neurologic comorbidities [3]. Future population-based studies would be instructive in determining the actual incidence of ANE in COVID-19. Similarly, the mechanism(s) driving ANE remain unknown. It is hypothesized that a 'cytokine storm' during systemic illness leads to blood-brain barrier breakdown, vascular endothelial injury, and cytotoxicity [15,16]. Elevated serum levels of tumor necrosis factor (TNF) and IL-6 are a typical feature of ANE, though CSF levels are less clear [15]. After SARS-CoV-2 was found to use the angiotensin-converting enzyme 2 (ACE2) receptor for cellular entry [17], which is expressed by neurons and glial cells, it was speculated that direct invasion of the CNS by SARS-CoV-2 could be pathogenic [18]. Interestingly, several investigators have detected SARS-CoV-2 RNA in the CSF of encephalitis patients, but it remains unclear whether this reflects direct infection of the CNS parenchyma or passive entry through a perturbed blood-brain barrier [16]. Another study did not find evidence of SARS-CoV-2 RNA in the CSF or cytokine storm in patients with neurologic complications of COVID-19, though that did not include cases of ANE [19]. Since many more people develop severe systemic inflammation from COVID-19 than go on to develop ANE, a multitude of factors likely influence the pathogenesis of ANE including genetics, age, and comorbidities. Indeed, the importance of genetic factors was reinforced by the discovery of mutations in the nuclear pore gene RANBP2 that cause familial and recurrent forms of ANE [15,20]. Further investigation into genetic factors that confer the risk of ANE may be helpful clinically, in theory allowing focused monitoring of and early intervention on patients with COVID-19 and genetic risk of ANE.

The case presented here fits many aspects of the clinicoradiologic syndrome ANE. The patient was found unresponsive after three days of COVID-19 symptoms, with imaging showing areas of T2 hyperintensity, diffusion restriction, and susceptibility artifact in the brainstem, medial temporal lobes, and thalami. This case is notable for the early onset of encephalitis in the clinical course and the relatively mild COVID-19 symptoms at the time of developing ANE. An important limitation of this report is the lack of CSF analysis for SARS-CoV-2 mRNA or antibodies. The patient initially experienced significant neurologic improvement after prompt immunosuppression using plasmapheresis and steroids, but ultimately succumbed to respiratory failure. This occurred despite stable hemodynamics and no sign of respiratory distress at discharge, and the cause of the rapid medical decline is unclear. An important possibility to consider is whether plasmapheresis or IV steroid treatment weakened the immune system, impairing its ability to combat SARS-CoV-2 and leading to worsened lung injury. While managing ANE and SAR-CoV-2, the risks of aggressive immunosuppression may need to be carefully weighed against the risk of uncontrolled infection. Additional studies are needed to understand the spectrum of ANE related to SARS-CoV-2 and long-term treatment outcomes.

## Conclusions

Here we present the case of a 33-year-old woman who developed ANE with rapid progression to coma the day after developing symptoms of SARS-CoV-2. She was treated rapidly with a combination of plasmapheresis and high dose IV corticosteroids. While she had neurologic improvement at the time of discharge, she died due to respiratory failure shortly thereafter.
